# Effect of hypobaric storage on quality, antioxidant enzyme and antioxidant capability of the Chinese bayberry fruits

**DOI:** 10.1186/1752-153X-7-4

**Published:** 2013-01-14

**Authors:** Hangjun Chen, Hailong Yang, Haiyan Gao, Jie Long, Fei Tao, Xiangjun Fang, Yueming Jiang

**Affiliations:** 1 Food Science Institute, Zhejiang Academy of Agricultural Sciences, 310021, Hangzhou, China; 2 School of Life & Environmental Sciences, Wenzhou University, 325035, Wenzhou, China; 3 South China Botanical Garden, Chinese Academy of Sciences, 510650, Guangzhou, China

**Keywords:** Chinese bayberries, Hypobaric storage, Quality, Antioxidant enzymes, Antioxidant capacity

## Abstract

**Background:**

The Chinese bayberry (*Myrica rubra* Sieb. and Zucc.) is a subtropical fruit native to China, with unique flavor, sweet and sour taste, and high nutrition and health values. The fruit is highly perishable and susceptible to mechanical injury, physiological deterioration and fungal decay once harvested. This study was to investigate the effect of hypobaric storage on the quality of Chinese bayberry fruit and then develop storage technology to prolong the supply of the fruit.

**Results:**

The fruit stored under hypobaric conditions exhibited lower decay, higher titratable acidity and total phenolics compared with those stored under normal atmospheric conditions. Hypobaric storage significantly reduced malonaldehyde accumulation, respiratory rate and maintained high catalase and peroxidase activities of Chinese bayberry fruit. Ferric reducing antioxidant power was also higher in the fruit stored under hypobaric condition than those under normal atmospheric conditions.

**Conclusion:**

Hypobaric storage improved the metabolism, antioxidant system and postharvest quality of Chinese bayberry fruit and provided an effective alternative method to prolong the storage life of this fruit.

## Background

The Chinese bayberries (*Myrica rubra* Sieb. and Zucc.) are a subtropical fruit native to China. In terms of the unique flavor, sweet and sour taste, attractive red color, and high nutrition and health values; Chinese bayberries have been cultivated in eastern and southern China for more than 2000 years and are being introduced to other countries. The fruit mature in early summer season and are praised as the “precious southern Yangtze fruit of early summer” [1-3]. The Chinese bayberries contain abundant anthocyanins, flavonoids and other phenolic compounds, with high antioxidant capacity [3-5]. Unfortunately, the fruit are highly perishable and susceptible to mechanical injury, physiological deterioration and fungal decay, resulting in a short postharvest life of 1−2 days at ambient temperature [6]. Some methods, including low temperature storage [7,8], high oxygen atmosphere treatment [9,10], hot air treatment [6,11], combined treatment of ethanol vapor with hot air [12], have been used to investigate postharvest physicochemical and physiological attributes and storage life extension of the Chinese bayberry fruit. However, due to the delicate nature of the fruit, poor handling practices and inadequate storage facilities, the shelf life of the Chinese bayberry is still short, which markedly limits its market. As this fruit is further commercially developed, it is important to develop effective storage methods to prolong the shelf life.

Among these techniques for controlling postharvest decay of fruit and vegetables, the use of sub-atmospheric pressure exhibits a potential to store fresh Chinese bayberries. Hypobaric storage can quickly remove heat and reduce oxygen level [13]. During storage, water spray could be used to solve the problem of insufficient environmental humidity [14]. It has been reported that hypobaric treatment delayed ripening of some climatic fruits such as apples, avocados, bananas, mangoes, tomatoes, apple, sweet cherry, asparagus, and peach [14-17]. With the development of storage technology, different models of hypobaric storage machine have been developed and tested for the storage of fruit and vegetables. However, little information is available in the literature about this storage technology for Chinese bayberry fruit.The objective of this present study was to investigate the effects of different hypobaric storage treatments on postharvest life and quality of the Chinese bayberry fruit. The antioxidant enzyme activities and antioxidant capacity were also evaluated. Finally, the optimal condition of hypobaric storage to extend the shelf life of the Chinese bayberry fruit was determined.

## Results and discussion

### Effect of hypobaric storage on fruit decay of Chinese bayberries

**Figure 1:**
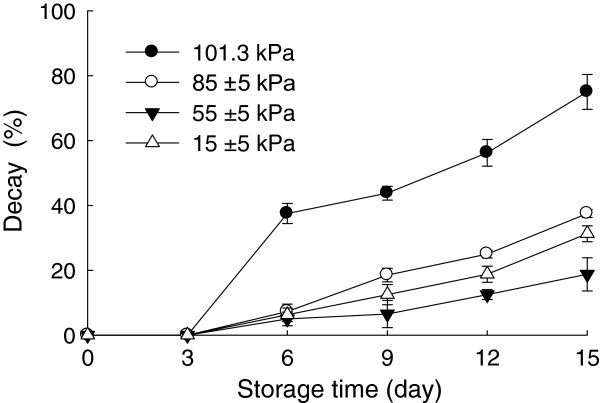
**Effects of hypobaric storage on fruit decay of the Chinese bayberry.** Fruits were stored at 1±0.5°C and 85–90% relative humidity under different atmosphere pressures.

Chinese bayberries are highly perishable and susceptible to mechanical injury, physiological deterioration and fungal decay [6]. The fruit stored under normal atmospheric pressure (control) showed 37.5% decay after 6 days of storage, but the fruit stored under 85±5, 55±5 and 15±5 kPa exhibited 7.25, 5.0 and 6.25% decay, respectively. As shown in Figure 1, the decay severity increased gradually with increasing storage. After 15 days of storage, the decay percentages of the Chinese bayberries stored under 101.3, 85±5, 55±5 and 15±5 kPa were 81.2, 31.25, 18.75 and 25%, respectively. It was reported that low pressure treatment discouraged commodity deterioration caused by bacteria and fungi and was capable of killing many insects infesting agricultural commodities [18]. Romanazzi, et al. [19] reported hypobaric treatment was effective in reducing decay of sweet cherries, strawberries and table grapes. The present study showed that hypobaric storage was an effective method to reduce decay of the Chinese bayberry. Among these four treatments, it was found that application of 55±5 kPa was the optimal to reduce the fruit decay.

### Effects of hypobaric storage on skin colour and pulp TSS and TA

**Figure 2:**
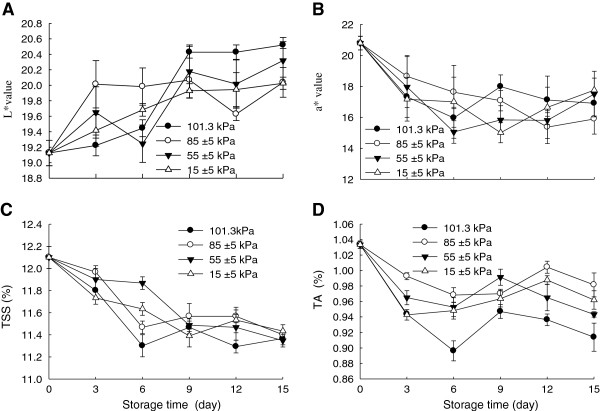
Effects of hypobaric storage on skin colour (A: L* value, B: a* value) and contents of total soluble solids (C) and titratable acidity (D) of the Chinese bayberry fruit. Fruits were stored at 1±0.5°C and 85–90% relative humidity under different atmosphere pressures.

The Chinese bayberry fruit of most cultivars are red or dark-red colour due to the presence of anthocyanins [3-5]. The major anthocyanin in the Chinese bayberry fruit was identified to be cyanidin-3-glucoside which represented more than 95% of the total anthocyanins [4]. Skin colour is an important index to evaluate the quality of the Chinese bayberry fruit. As shown in Figure 2A and 2B, L* value increased while a* value of the fruit decreased gradually during storage. However, no significant (P<0.05) differences in the skin colour were observed among these different pressure treatments after 15 days of storage.

**Figure 3:**
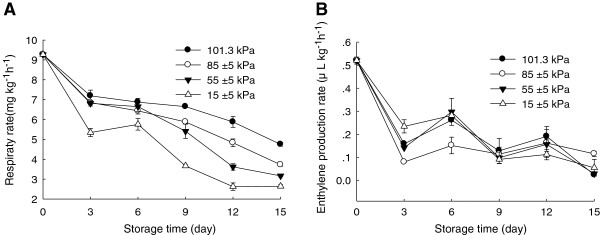
**Effects of hypobaric storage on rates of respiration (A) and ethylene production (B) of the Chinese bayberry fruit.** Fruit were stored at 1±0.5°C and 85–90% relative humidity under different atmosphere pressures.

TSS content of the Chinese bayberry fruit decreased during storage (Figure 2C) but no significant (P<0.05) differences existed among the different pressure treatments. Figure 2D presented TA content of the Chinese bayberries. The TA content decreased gradually during storage. By the end of storage, TA content decreased to 11.6, 5.0, 8.7 and 6.9% under 101.3, 85±5, 55±5 and 15±5 kPa conditions, respectively. The changes in TA and TSS contents could be associated with the metabolic activity and respiratory rates of the fruits. Corey, et al. [20] reported that respiration rate of lettuce decreased by 40% when stored in a chamber at a pressure of 51 kPa. The results in this study could be explained by a lower respiratory rate (Figure 3A), which caused less depletion of sugars and acids when fruit was stored at lower pressures.

### Effects of hypobaric storage on respiration and ethylene production rates

The respiratory rate of the Chinese bayberry fruit was around 9 mg CO_2_ kg^-1^ h^-1^ before storage and decreased gradually with increasing storage (Figure 3A). By the end of storage, respiratory rates of the Chinese bayberry stored under 101.3, 85±5, and 55±5 kPa conditions were 4.75, 3.72, and 3.16 mg CO_2_ kg^-1^ h^-1^, respectively. The respiratory intensity of the fruit under hypobaric storage was significantly (P<0.05) inhibited, as compared with under normal atmospheric pressure. Ethylene production rates of the Chinese bayberries decreased gradually, from 0.52 before storage to 0.042 μL kg^-1^ h^-1^ by the end of storage (Figure 3B). However, no significant differences in ethylene production rates were observed between the hypobaric storage and normal storage.

It was reported that hypobaric packaging reduced the respiration rates of strawberry and curled lettuce [21]. He et al. [22] also reported that hypobaric storage conditions could reduce greatly the ethylene production rate in both lettuce and wheat. The removal of ethylene production could delay senescence of fruits and vegetables and, indirectly, reduce their susceptibility to pathogens [19]. The inhibition of the respiratory rate in the Chinese bayberry fruit by hypobaric treatment can help to extend the shelf life.

### Effect of hypobaric storage on malondialdehyde (MDA) content

**Figure 4:**
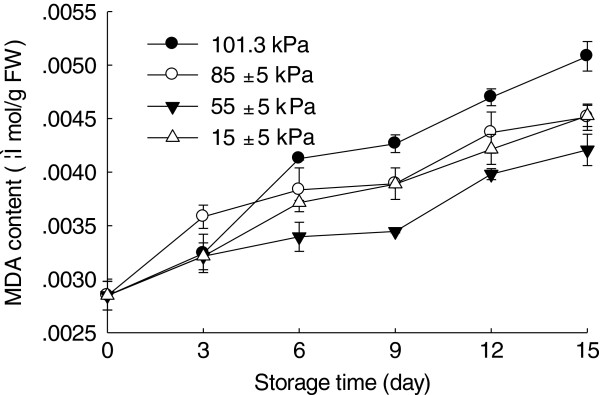
**Effects of hypobaric storage on MDA content of the Chinese bayberry fruit.** Fruit were stored at 1±0.5°C and 85–90% relative humidity under different atmosphere pressures.

MDA is considered to be an indicator of membrane lipid peroxidation caused by oxidative stress. As shown in Figure 4, MDA content of the Chinese bayberry fruit under normal pressure condition rose gradually during storage. MDA contents of fruit stored under 101.3, 85±5, 55±5 and 15±5 kPa conditions after 15 days of storage were 5.08×10^-3^, 4.51×10^-3^, 4.21×10^-3^ and 4.53×10^-3^ μmol g^-1^ on fresh weight (FW) basis, respectively, which exhibited that hypobaric storage inhibited the accumulation of MDA. Similar results were obtained by Li et al. [14] who reported that hypobaric storage could reduce MDA accumulation and retard senescence in asparagus.

### Effect of hypobaric storage on antioxidant enzyme activities

**Figure 5:**
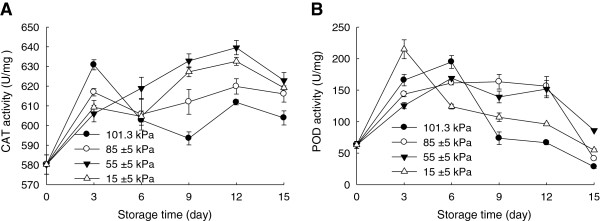
**Effects of hypobaric storage on activities of CAT (A) and POD (B) of the Chinese bayberry fruit.** Fruit were stored at 1±0.5°C and 85–90% relative humidity under different atmosphere pressures.

The accumulation of reactive oxygen species, such as superoxide, hydrogen peroxide, and the hydroxyl radical, causes plant tissue damage and reduces the storage quality and marketability of fruits and vegetables [23]. Antioxidative enzymes such as catalase (CAT) and peroxidase (POD) play an important role to scavenge reactive oxygen. As shown in Figure 5A and 5B, CAT activities of the Chinese bayberry fruit tended to change differently at various pressure conditions, but the fruit stored under the hypobaric condition exhibited a higher activity than those stored under the normal pressure condition. For POD activity, hypobaric storage condition maintained a significantly (P<0.05) higher activity compared with the normal atmospheric condition. Chen et al. [17] reported that hypobaric storage maintained high CAT activity in peach. In this study, application of hypobaric storage enhanced CAT and POD activities and, thus, reduced membrane lipid peroxidation of the Chinese bayberry (Figure 4).

### Effects of hypobaric storage on total phenolic content and total antioxidant capacity

**Figure 6:**
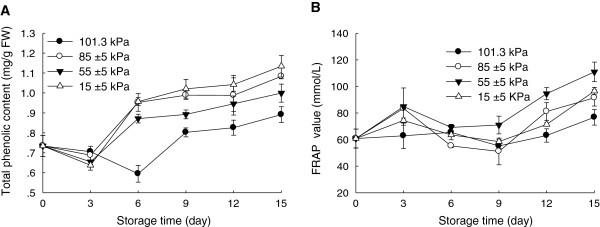
**Effects of hypobaric storage on total phenolic content (A) and total antioxidant capacity (B) of the Chinese bayberry fruit.** Fruit were stored at 1±0.5°C and 85–90% relative humidity under different atmosphere pressures.

Phenolic compounds including flavonoids and phenolic acids are known to be responsible for antioxidant capacity in fruits. The Chinese bayberries are rich in phenolic compounds and exhibit high antioxidant activity [3,4,24]. As shown in Figure 6A, total phenolic content of the fruit increased during storage. However, no significant differences were observed among these different pressure treatments within the first 3 days of storage. The total phenolic content of the fruit under the hypobaric conditions after 3 days of storage rose rapidly, and then reached 1.083, 0.999, and 1.134 mg g^-1^ FW when fruit were stored at 85±5, 55±5 and 15±5 kPa after 15 days of storage, respectively, but only 0.892 mg g^-1^ FW at 101.3 kPa, which indicated that hypobaric storage was more effective to maintain phenolic content of the fruit. In similarity with the change in total phenolic content, reducing power of fruit rose gradually during storage (Figure 6B). In this study, the fruit stored under 55±5 kPa showed the strongest ferric reducing antioxidant power. However, ferric reducing antioxidant power (FRAP) was not correlated well with the total phenolic contents. It has been reported that different antioxidant activity could be due to the difference in phenolic constituents [25].

## Materials and methods

### Fruit materials

The Chinese bayberries (*Myrica rubra* Sieb. & Zucc., cv. Dongkui) fruit were harvested manually from a commercial orchard in Xianju county of Zhejiang Province, China on June 28, 2010, and then transported to the laboratory by a refrigerated car within 3 hours. Fruit were selected for uniformity of shape and color and the blemished and diseased fruit were discarded.

### Fruit treatments

Fruit treatments were performed in a hypobaric storage system with storage chambers whose pressure could be set independently (Model XL-5, Xianlv Low-pressure Fresh Keeping Equipment Co. Ltd., Shanghai, China). Each replicate containing 2 kg fruits was put in a plastic basket and placed into the hypobaric chamber. The applied pressures were set to be 85±5, 55±5 and 15±5 kPa, respectively. The normal atmospheric pressure (101.3 kPa) was used as control. These fruit were stored at 1±0.5°C and 85–90% relative humidity (RH). Fruit samples were taken for analysis every 3 days in the storage period of 15 days.

### Evaluations of fruit decay and skin colour

Fruit decay was visually evaluated. Fruit with visible mold growth with about 2% of the surface affected was considered rotten. The severity of fruit decay was expressed as percentage of fruit showing decay symptoms. Skin colour of 20 fruit from each replicate was measured using a colorimeter (Konica Minolta, CR-400, Japan) with a 6-mm aperture size, which provided L* and a* values according to the system established by the Commission Internationale de L’Eclairage (CIE, International Commission on Illumination). A reference white tile was used for calibration.

### Measurements of respiration and ethylene production rates

Ten fruit were enclosed in 250 mL glass jars at 5°C for 2 h and then 2 mL of headspace gas were taken from each jar. CO_2_ amount was measured by gas chromatography (Rainbow, SP-9890, China) equipped with flame ionization detector and a packed column (GDX-502, Zhonghuida Inc., China). Ethylene concentration was analysed by gas chromatography using a flame ionization detector. Respiration and ethylene production rates were expressed as mg CO_2_ and μg per hour on fresh weight basis, respectively.

### Measurements of total soluble solids and titratable acidity

Fifty fruit from each treatment were taken. Juice was obtained by a juicer (HR1861, Philips Co. Beijing, China), followed by filtration through cheesecloth. The juice was analyzed for total soluble solids (TSS) and titratable acidity (TA). TSS concentration was determined by a portable refractometer (Atago PAL-1, Japan) while TA content was measured by titrating 20 mL of the juice to pH 8.2 using 0.1 mol L^-1^ NaOH.

### MDA content determination

MDA content was determined according to the method described by Li et al. [14] with some modification. Fruit tissues (1 g) were extracted for 2 h with 5 mL of trichloroacetic acid (10%). Three milliliters of 0.5% thiobarbituric acid (TBA) in 10% trichloroacetic acid were added to 1 mL of the extract. The solution was heated in a boiling water bath for 20 min, then immediately cooled, and finally centrifuged at 6000 × g for 10 min to clarify the solution. Absorbance was measured at 532 and 600 nm. MDA content was expressed as μmol/g FW by the method of Li et al. [14].

### Enzymatic activity assay

Five grams of fruit tissues were homogenized in 25 mL of 100 mmol L^-1^ Tris-HCl buffer (pH 7.8) containing 2 mmol L^-1^ EDTA and 2 mmol L^-1^ 1,4-dithiothreitol at 4°C. The homogenate was centrifuged at 15,000 × g for 15 min at 4°C, and then the supernatant was collected for the enzymatic activity assay. Protein was measured according to the method of Bradford [26], using bovine serum albumin (BSA) as the standard.

CAT was analyzed according to the method of Beers & Sizer [27] with some modifications. The disappearance of H_2_O_2_ was monitored by measuring the decrease in absorbance at 240 nm of a reaction mixture containing 100 mmol L^-1^ Tris-HCl buffer (pH 7.8), 25 mmol L^-1^ H_2_O_2_, and 0.2 mL of crude enzyme extract. One unit of enzymatic activity was defined as 0.01 change of absorbance at 240 nm per minute. Specific CAT activity was expressed as units per mg protein. POD activity was assayed according to the method described by Yang et al. [10]. The reaction mixture (2 mL) consisted of 50 mmol L^-1^ sodium phosphate buffer (pH 6.5), 6 mmol L^-1^ guaiacol and 4.5 mmol L^-1^ H_2_O_2_ prior to the addation of 1 mL of crude enzyme extract. Increase in absorbance at 470 nm at intervals of 30 s was recorded. One unit of enzymatic activity was defined as the amount of enzyme that catalyzed the peroxidation of 1 mmol of guaiacol per minute. Specific POD activity was expressed as units per mg protein.

### Total phenolic content determination

One gram of lyophilized fruit tissues was extracted with 25 mL of ethanol for 3 h. Total phenolic contents were estimated colourimetrically using the Folin-Ciocalteu method [28]. The extract was appropriately diluted, and then 1 mL of the dilution was oxidized with 0.5 mL of Folin-Ciocalteau reagent. The reaction was neutralized with 5 ml of 5% Na_2_CO_3._ The solution was immediately diluted to a final volume of 25 mL with distilled water and then mixed thoroughly. The absorbance was read at 765 nm after 1 hour of incubation in dark at 25°C using a spectrophotometer (Shimadzu UV-2550, Japan). Gallic acid was used as a standard, and phenolic contents were expressed as mg gallic acid equivalents (GAE)/g FW.

### FRAP assay

The ferric reducing ability of the Chinese bayberry was measured according to the method of Benzie & Strain [29]. To prepare the FRAP reagent, a mixture of 0.3 mol L^-1^ acetate buffer (pH 3.6), 10 mmol L^-1^ tripyridyltriazine (TPTZ), and 20 mmol L^-1^ ferric chloride (10:1:1, v/v/v) was made. One gram of lyophilized fruit tissues was extracted for 12 hours with 20 mL of ethanol. The FRAP reagent (3.9 mL) was added to the extract solution sample (0.1 mL) and then mixed thoroughly. The reaction was then monitored for 10 min at 37°C and the absorbance was recorded at 593 nm on the Shimadzu UV-2550 spectrophotometer. The ferric reducing ability of the Chinese bayberry fruit was expressed as mmol FeSO_4_ per litre crude extract.

### Data analysis

All samples were prepared and analysed in triplicate. Statistical analysis was done with one-way analysis of variance using the SAS statistical software package.

## Conclusions

Previous studies indicated that hypobaric storage reduced commodity respiration and prevented wilting and senescence during storage [17,30]. In the present study, application of hypobaric pressure to the Chinese bayberries significantly reduced fruit decay and loss in total acids, inhibited respiratory rate, decreased MDA accumulation and maintained total phenolic content, antioxidant capacity and CAT and POD activities. These data suggested that hypobaric storage could be an effective technology in maintaining postharvest quality and prolonging shelf life of Chinese bayberry fruit.

## Competing interests

The authors declare that they have no competing interests.

## Authors’ contributions

HC made a significant contribution to acquisition of data, and data analysis. HY made a substantial contribution to data analysis and manuscript preparation. HG made a significant contribution to experimental design and data analysis. JL made a contribution to acquisition of data, and data analysis. FT and XF participated in some experiments. YJ made a significant contribution to experimental design, data analysis and manuscript revision. All authors read and approved the final manuscript.
